# Protocol for a prospective cohort study on congenital heart disease in neonates across varied altitudes in Sichuan province, China

**DOI:** 10.1371/journal.pone.0319709

**Published:** 2025-04-16

**Authors:** Xianmin Wang, Jiaxin Liu, Wen Su, Beibei Liu, Lixing Li, Duo Yan, Weiyi Wan, Tongyong Luo

**Affiliations:** 1 Pediatric Cardiology Center, Sichuan Provincial Women’s and Children’s Hospital, Affiliated Women’s and Children’s Hospital of Chengdu Medical College, Chengdu, Sichuan, China; 2 Department of Population Care, Sichuan Provincial Women’s and Children’s Hospital, Affiliated Women’s and Children’s Hospital of Chengdu Medical College, Chengdu, Sichuan, China; 3 School of Public Health, Chengdu Medical College, Chengdu, Sichuan, China; Sreenidhi Institute of Science and Technology, India

## Abstract

Congenital heart disease (CHD) is the most prevalent congenital disorder, contributing significantly to neonate mortality. Despite advances in diagnosis and treatment, the incidence and risk factors of CHD remain underexplored, particularly in regions with varied altitudes. Sichuan Province, China, with its diverse topography and altitudes, provides a unique opportunity to investigate the epidemiology of CHD across different environmental settings. This study aims to explore the incidence, subtypes, and risk factors of CHD in neonates across high, middle, and low-altitude regions of Sichuan Province. It also seeks to assess the effectiveness of the Sichuan Province Newborn CHD Free Screening Project, the impact of CHD on family economics and child development, and to provide data-driven recommendations for improving CHD prevention and control measures. We will conduct a multicenter, prospective cohort study involving neonates with positive CHD screening results and their families, recruited from three cities representing different altitude levels: Aba Tibetan and Qiang Autonomous Prefecture, Mianyang City, and Guangyuan City. Data collection will include birth characteristics, CHD screening outcomes, parental and gestational histories, and blood samples for genetic analysis. The study will monitor treatment outcomes, economic impact, and the growth and development of the children over time. This study will provide critical insights into the epidemiology of CHD in Sichuan Province, particularly in relation to altitude. The results will help optimize CHD screening and management programs, ultimately improving outcomes for affected children and their families.

## Background

Congenital heart disease (CHD) is the most prevalent congenital disorder in newborns and a leading cause of infant mortality globally. The incidence of CHD varied from 8 to 16 per 1000 live births, depending on the various social development index of the country[1–3]. In China, recent studies indicate a similar burden with specific regional variations impacting disease prevalence and outcomes[4].

The development of CHD is influenced by a complex interaction of genetic and environmental factors. Genetic Factors, including genetic mutations, chromosomal abnormalities, and intricate inheritances are associated with CHD[5]. Syndromes like Down syndrome, Turner syndrome, and 22q11.2 deletion syndrome are frequently linked with CHD[6]. Single-gene mutations, seen in conditions such as Marfan syndrome and Holt-Oram syndrome, can also result in syndromic CHD[7]. Studies revealed that CHD prevalence varies among different ethnicities, highlighting the role of genetic factors[8, 9]. Modern techniques such as next-generation sequencing are increasingly used to identify subtle genetic variations that contribute to CHD, helping in understanding the molecular pathways involved in cardiac development[5]. Maternal illnesses, for example, maternal diabetes mellitus, phenylketonuria, and viral infections like rubella during pregnancy are strongly associated with an increased risk of CHD in the offspring, affecting fetal heart development during critical periods of pregnancy[10, 11]. Certain medications, for example, antiepileptic drugs like phenobarbital and valproic acid have been associated with cardiac malformations[12]. Poor maternal nutrition is associated with CHD, particularly deficiencies in critical nutrients like folic acid, which is essential for proper neural tube closure and cardiac development[13–15]. Lifestyle Factors, including smoking and alcohol consumption during pregnancy have been linked to a higher incidence of CHD. Nicotine and alcohol can interfere with fetal development by limiting oxygen and nutrient supply to the fetal tissues, including the developing heart[16–18].

Emerging research suggests that high altitude might be a specific environmental risk factor for CHD[8,19,20]. Individuals living at high altitudes exposes to hypoxic conditions, which could impact placental function and fetal oxygenation. Studies have shown a higher incidence of CHD in populations living at high altitudes in Ecuador[21], Colombia[8,20], China[19,20,22], potentially due to chronic hypoxia on fetal cardiac morphogenesis. For instance, a meta-analysis revealed that the prevalence of CHD among individuals living above 1,500 meters was 8.97%, with atrial septal defect being the most common type[23]. This hypothesis is supported by findings that chronic hypoxia can alter gene expression and disrupt the normal signaling pathways necessary for cardiac morphogenesis. High-altitude areas have strong ultraviolet radiation and reduced oxygen supply, which may more significantly affect the development of the cardiovascular system in newborns compared to plain areas, further impacting the incidence of CHD.

The mechanisms by which these risk factors contribute to CHD are complex and multifaceted. Genetic factors may predispose to structural abnormalities during cardiac development, while environmental influences can exacerbate these risks or independently affect cardiac morphology. Hypoxia, oxidative stress, and disruptions in the maternal immune environment are potential pathways through which environmental factors like smoking, alcohol, and maternal illnesses may influence cardiac development in the fetus.

Sichuan Province is located in southwestern China, featuring complex and varied terrain. It borders the Qinghai-Tibet Plateau to the west and the Chengdu Plain to the east, with a terrain that slopes downward from west to east. The average altitude is 2522 meters, with the highest being 6536 meters and the lowest 140 meters. By 2020, Sichuan was home to a diverse population, with of 568.8 million ethnic minority people, making up 6.8% of the total population. These minorities are distributed throughout the province in various forms of habitation. A paper published the CHD incidence rate of 2.18% (3285/150,983) in Chengdu, the capital of the Sichuan and its surrounding areas from 1988 to 2004[24], another paper published in 2013, CHD incidence rate of 0.68% (68/10,021) in Liangshan Prefecture, with the top three subtypes diagnosed being atrial septal defects, patent ductus arteriosus, and ventricular septal defects[25]. However, our literature review indicates that research on the epidemiological status of CHD in Sichuan province is scarce and lacks systematization.

The Sichuan Province Newborn CHD Free Screening Project (Free Screening Project) has been set up from the end of 2023. Pulse Oximetry and Auscultation of heart murmur are applied to the CHD screening for neonate in 6–72 hours. Neonates with Oxygen saturation less than 95% or difference more than 3% in both limbs, or ≥ Grade 2 of Heart murmur will be positive results. Echocardiography (ECG) is applied to the positive screening neonate diagnosis. Our study will build on this “Free Screening Project” to understand the incidence of CHD in different altitude regions of the province, by further to identify common and specific risk factors affecting the incidence of CHD different cities (prefectures), examine post-diagnosis treatment conditions, subsequent quality of life of the children, and the economic burden on families, thereby providing references for the precise prevention and control of CHD throughout the province.

## Methods and analysis

### Study design

This multicenter prospective cohort study will be conducted. Eligible participants include neonates with positive preliminary screening results for CHD through Sichuan Province’s free neonatal screening program, as well as their families. Key data to be collected include birth characteristics, CHD screening and diagnostic outcomes across cities with varying altitudes. Demographic factors and potential risk factors will be assessed using standardized questionnaires. The study will also track treatment outcomes, changes in family economics, and the growth and development of the neonates. Additionally, blood samples will be collected for subsequent genetic analyses.

### Research sites

Three cities have been selected to represent the high, middle, and low altitude regions in Sichuan Province:

- **Aba Tibetan and Qiang Autonomous Prefecture**: Average altitude 3542 meters, ranging from 449 to 6007 meters. About 5,000 live neonates in 2023.
- **Mianyang City**: Average altitude 1392 meters, ranging from 265 to 5390 meters. About 23,000 live neonates in 2023.
- **Guangyuan City**: Average altitude 805 meters, ranging from 439 to 1676 meters. About 12,000 live neonates in 2023.

### Study participants

- Inclusion criteria: Neonates with positive result of screening in the Free CHD Screening Program and their families will be recruited. Neonates with positive CHD screening results will be identified using pulse oximetry (SpO2 <95% or >3% difference between limbs) and auscultation (Grade ≥2 heart murmur).
- Exclusion criteria: Families without smart devices or those with mental disorders preventing independent completion of the survey.

*The project recruitment staff will inform the guardians of CHD screening positive neonates who meet the inclusion and exclusion criteria about the project. After fully understanding the significance of the project, their rights and obligations, and voluntarily signing the informed consent form, they will be included as research subjects for this project.*

### Sample size estimation

Utilizing birth data from 2022 across the three cities/prefectures in Sichuan, and assuming a CHD incidence rate of approximately 1%, adjustments account 75% for exclusion criteria, consent limitations, and potential dropouts to ensure statistical robustness, it is projected that the regions will recruit approximately 300 CHD-affected neonates annually. We hope to establish a cohort of more than 900 participants between 1st January 2024 and 31st December 2026.

### Research questions

- What are the incidence rates and subtypes of CHD among neonates in the three cities?
- How do these rates and subtypes differ among the cities?
- If differences exist, which risk factors contribute to these variations, and does altitude play a role as a potential risk factor for CHD?
- What is the positive rate of CHD screening and diagnosis, and is there any variation among the three cities? If so, what factors contribute to these differences?
- What interventions are provided to diagnosed CHD children in the three cities?
- What is the cost-effectiveness of the free screening program in the three cities?
- Are there any differences in the growth and development of children among the three cities, and does CHD impact this?
- What recommendations can be made to the government to enhance the effectiveness of the free screening program?

### Study procedure

#### Study participant recruitment.

Families of neonates with positive CHD screening results will be recruited in the screening units after signing a consent form to participate in the study. Data will be collected using the following questionnaire.

#### Data collection.

- Number of neonates screened, positive screening results, positive diagnoses, and types of CHD. CHD cases will be categorized by severity using American Heart Association (AHA) guidelines.
- To ensure consistency, diagnostic thresholds will remain constant across all study sites, and all investigators will receive uniform training on screening and diagnostic protocols. Regular inter-laboratory validations will ensure diagnostic accuracy and consistency.
- Birth outcomes of neonates: sex, height, weight, health status, screening and diagnostic results, etc.
- Parental data: demographic information, CHD history, lifestyle factors (e.g., smoking, alcohol consumption).
- Gestational data of the mother: antenatal infections and medication history, occupational and environmental exposures, air pollution exposure, dietary and nutritional supplementation intake. Additionally, the main city where the pregnant woman resided during pregnancy will be recorded to adjust for altitude.
- Blood samples from neonates will be collected for further genetic research.
- To protect participant privacy, all collected data will be de-identified prior to deposition in a public repository. Data collection and sharing will adhere to the ethical standards outlined in the Declaration of Helsinki and the Regulations on Ethical Review of Life Sciences and Medical Research Involving Humans (China)

#### Identification of CHD incidence and risk factors.

- After ECG diagnosis, the positive rate of screening, incidence of CHD in neonates, and the accuracy rate of screening will be calculated.
- Subtypes of CHD will be documented
- Common and specific risk factors will be analyzed

#### Cost-effectiveness analysis.

- Confirmed CHD neonates will be managed based on the severity of their condition through regular follow-ups, medication, interventional treatment, or surgical procedures.
- Surgical outcomes, costs, and changes in the economic status of CHD neonate families will be collected.
- Growth and development of children, as well as quality of life, will be collected and analyzed.
- The cost-effectiveness analysis will assess screening costs per detected CHD case, stratified by region and altitude. Metrics include direct medical costs, long-term care expenditures, and health-related quality of life (HRQoL) scores.

#### Strategies for improving the free project.

Based on the findings, strategies will be suggested to improve the effectiveness and efficiency of the free screening program.

**Fig 1:**
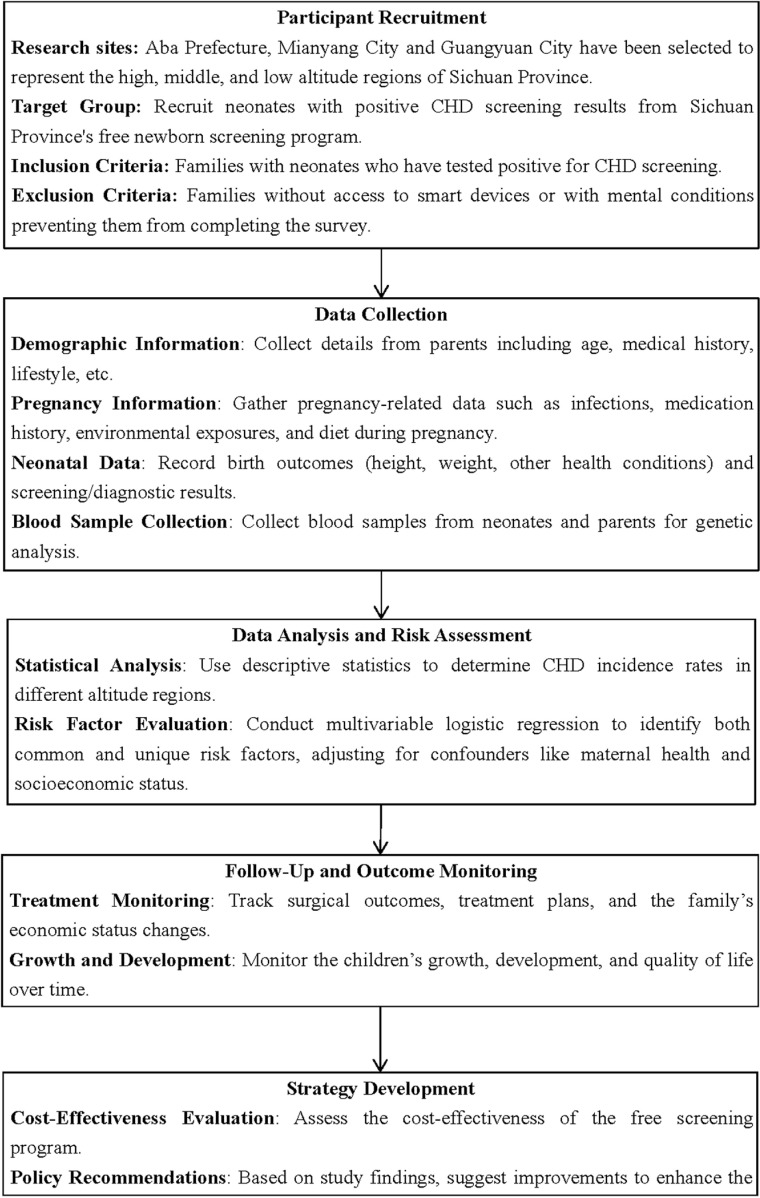
Flowchart of study procedure.

An overview of the study design, including participant recruitment, screening procedures, diagnostic confirmation, data collection, and follow-up assessments can be seen in the Flowchart of the study procedure (Fig 1).

### Analysis plan

Descriptive statistics will be utilized to characterize the study cohort. Multivariable logistic regression analysis will identify risk factors associated with CHD, with adjustments made for potential confounders such as maternal health and socioeconomic status. Comparative analyses will evaluate variations in CHD incidence rates across different altitudes within Sichuan Province and between the province and the specific study regions. The study will also examine common and unique risk factors influencing CHD incidence at varying altitudes. Additionally, treatment conditions, family economic changes, and life quality of post-diagnosis will be compared between the CHD group and the control group. Bias Mitigation: Potential biases, such as diagnostic inconsistencies across altitudes, will be minimized through standardized training of investigators and consistent application of diagnostic criteria across all sites.

### Key points for study quality

The primary focus of this study is follow-up surveys, which involve multiple, lengthy, and repeated investigations, encompassing various survey contents and multiple types of questionnaires. Therefore, the quality of the survey design, the conduct of follow-up surveys, and the low dropout rate of the study cohort will determine the overall quality of this research. To enhance the quality of the investigation, the study will address the following aspects:

- Survey Design and Quality: Develop comprehensive and user-friendly questionnaires that cover risk factors, growth indicators, and assessments of quality of life.
- Investigator Training: Provide detailed training to investigators to ensure consistent recruitment and data collection methods across different regions.
- Participant Engagement: Promote the significance of the project, offer medical and child health consultations, and build trust with participant families to ensure sustained engagement.
- Assumptions underlying the analysis include uniformity in the application of CHD diagnostic criteria across altitudes and adherence to follow-up schedules over three years. Potential variability in data collection is mitigated through centralized training programs.
- To minimize bias, all investigators will undergo standardized training on data collection procedures. Diagnostic thresholds and protocols will be consistently applied across study sites to ensure reliability. A centralized database with restricted access will prevent variability in data handling

### Ethical approval and consent to participate

This study has been approved by the Medical Ethics Review Committee of the Sichuan Provincial Women’s and Children’s Hospital, with the approval number 20231102–294. The guidelines mentioned for adherence include the *Regulations on the Ethical Review of Life Sciences and Medical Research Involving Humans*“ (China), the *Declaration of Helsinki (WMA)*”, and the “*International Ethical Guidelines for Biomedical Research Involving Human Subjects (CIOMS)*. *Informed Consent Form* for study participant with statements on consent to participate and consent to publish are also approved by the Review Committee as well.

## Discussion

Although reduced oxygen levels at high altitudes and increased ultraviolet radiation leading to genetic mutations are recognized as primary risk factors for CHD, additional factors, such as ethnic (genetic) factors and socioeconomic factors may also influence the incidence of CHD in the three study regions. Aba Prefecture is home to a significant proportion of the Tibetan and Qiang populations, with these ethnic groups comprising 82% of the local population. In contrast, Mianyang City and Guangyuan City have predominantly Han Chinese populations, with minorities scattered throughout. Previous studies have shown that CHD prevalence varies among different ethnicities, suggesting a role for genetic factors. Therefore, it is crucial to consider these ethnic differences in the analysis. The three regions exhibit varying socioeconomic statuses, which can affect prenatal care and maternal health, potentially contributing to the incidence of CHD. Lower socioeconomic status is often associated with poorer health outcomes and may be a confounding variable in the incidence of CHD. Given these considerations, the study will employ stratified analysis and multivariable analysis to identify the true risk factors influencing CHD incidence across the three regions. These statistical methods will help disentangle the effects of altitude, ethnicity, and socioeconomic factors on CHD incidence.

Numerous studies have suggested that the screening SpO2 thresholds should vary between high-altitude and low-altitude settings due to physiological adaptations to reduced oxygen availability. Typically, a lower threshold (such as an SpO2 value below 95%) is employed in high-altitude regions to minimize false-positive results, enhance specificity, and optimize the cost-effectiveness of the screening process. The relationship between high altitude and increased CHD prevalence underscores the importance of tailored screening programs in these regions. Research has shown that high-altitude environments are associated with a higher incidence of CHD, particularly atrial septal defects[26, 27].This adjustment helps to reduce unnecessary follow-up examinations and the associated workload for healthcare providers. Additionally, the effectiveness of pulse oximetry screening may vary with altitude, necessitating the development of altitude-specific screening algorithms[28]. In this study, we will evaluate the accuracy and cost-effectiveness of SpO2 screening across the three regions under investigation. If the findings indicate a need for adjustments, particularly regarding the screening threshold, we will promptly propose modifications to ensure that the screening process is both efficient and effective. Such adjustments will be informed by our comprehensive analysis of the data collected, taking into account the unique characteristics of each region, including altitude, ethnicity, and socioeconomic status. By carefully considering the specific needs of each region, this study aims to provide valuable insights into the optimal implementation of SpO2 screening for CHD in diverse settings, ultimately contributing to improved clinical guidelines and public health strategies.

### This study has some limitations

This study is the first to systematically to investigate CHD incidence and risk factors across Sichuan Province’s diverse attitudes. Although the selected regions provide representative data on altitude and population demographics, they may not fully represent the entire provincial context, which could introduce potential sampling bias. Furthermore, the estimated sample size of 900 participants over three years may be relatively small, which could limit the generalizability of the findings. Efforts will be made to secure additional funding post-study to expand the cohort and extend the study duration. The current protocol does not include genetic testing, relying instead on ethnic characteristics to infer potential genetic differences. Future work will seek funding to conduct genetic analyses on stored samples to explore the genetic basis of CHD more comprehensively. Despite these limitations, the study is designed to provide valuable insights into the epidemiology of CHD in Sichuan Province and to inform future preventive measures and public health policies.
